# Surveillance of Extrapulmonary Nontuberculous Mycobacteria Infections, Oregon, USA, 2007–2012

**DOI:** 10.3201/eid2310.170845

**Published:** 2017-10

**Authors:** Emily Henkle, Katrina Hedberg, Sean D. Schafer, Kevin L. Winthrop

**Affiliations:** Oregon Public Health Division, Portland, Oregon, USA (E. Henkle, K. Hedberg, S.D. Schafer);; Oregon Health and Science University, Portland (E. Henkle, K.L. Winthrop)

**Keywords:** Public health surveillance, incidence, nontuberculous mycobacteria, Mycobacterium avium complex, extrapulmonary nontuberculous mycobacteria, bacteria, Oregon, tuberculosis and other mycobacteria

## Abstract

Limited data are available describing extrapulmonary nontuberculous mycobacteria (NTM) infections in the general population. We describe results from statewide population-based laboratory surveillance in Oregon, USA, during 2007–2012. We defined a case of extrapulmonary NTM infection as >1 isolate from skin/soft tissue, disseminated sites, lymph node, joint, or other sites. The annual incidence of extrapulmonary NTM infection (other than *Mycobacterium gordonae*) was stable, averaging 1.5 cases/100,000 population. Median age of the 334 patients was 51 years, and 53% of patients were female. Half of cases were caused by *M. avium* complex, but rapid-growing NTM species accounted for one third of cases. Most extrapulmonary NTM infections are skin/soft tissue. Compared with pulmonary NTM infection, more extrapulmonary infections are caused by rapid-growing NTM species. the designation of NTM as a reportable disease in Oregon in 2014 will result in better detection of changes in the incidence and patterns of disease in the future.

Nontuberculous mycobacteria (NTM) are ubiquitous in water and soil and are a cause of opportunistic pulmonary and extrapulmonary infections. Extrapulmonary manifestations include disseminated, skin, joint, and lymph node infections. Extrapulmonary NTM infections are typically sporadic but may be associated with nosocomial outbreaks (*1*), clinical procedures (*2*), or nail salon pedicures (*3*). Treatment is species dependent, typically consisting of 3–6 months of multidrug antimicrobial therapy (*4*). Although numerous case series are found in the literature, few data exist to describe the population-based epidemiology of extrapulmonary NTM infections. Recent studies have shown an increase in the prevalence and incidence of pulmonary infections (*5*,*6*). We describe the results of a statewide laboratory surveillance study in Oregon, USA, that identified all patients from whom NTM was isolated from extrapulmonary sites during 2007–2012. We report characteristics of patients with extrapulmonary NTM infection by species and site and calculate the annual incidence over the study period.

## Methods

Methods have been described elsewhere (*5*). In brief, we requested positive NTM culture results from January 1, 2007, through December 31, 2012, from all 17 laboratories that perform acid-fast bacillus culture in Oregon or are used as reference laboratories. We excluded *M. tuberculosis* and *M. bovis*. Additional data for each positive culture included patient name, age at collection or date of birth, address or county and ZIP code of residence, species isolated, date of collection, and body source of isolate. One laboratory in central Oregon was unable to provide enough information to assign a state of residence. Given a low likelihood of these patients coming from out of state (this lab is >120 miles from Washington, Idaho, or California), however, we considered all these patients to be Oregon residents. After we identified the patients, we linked patients with extrapulmonary isolates to the state HIV database to identify any who were infected with HIV.

## Case Definition

We defined an extrapulmonary NTM infection case as having >1 isolates from skin/soft tissue (wound, abscess, tissue, or exit catheter); disseminated sites (blood, bone marrow, cerebrospinal fluid, pericardial fluid, or peritoneal fluid); lymph node (lymph node or neck abscess); joint (synovial or joint fluid); or other (urine, eye, sinus, or nasopharyngeal). We excluded isolates from an unknown source or from feces, saliva, or gastric sites. *M. gordonae* was reported but is considered nonpathogenic, so we excluded it from estimates of disease incidence. Rapid-growing mycobacteria (RGM) species include *M. chelonae/abscessus* complex, *M. fortuitum*, and *M. chelonae* (*4*); we included these in the analysis.

## Statistical Analysis

We described patients by age, sex, and species isolated overall and by source of specimen. We considered extrapulmonary cases to be incident at the time of isolation, given that treatment for extrapulmonary infections is typically in the range of weeks to several months and the cure rate is high (*4*). We calculated the annual incidence as the number of new cases in a given year divided by the midyear population using population data from the Portland State University Population Research Center (*7*) and report the average annual incidence and standard 95% CIs for 2007–2012. We used Poisson models using a log link to estimate the overall incidence rate trend over the study period. We imported all data into SAS version 9.3 (SAS Institute Inc., Cary, NC) for analysis. The study was considered to be public health practice (nonresearch) by the Oregon Health Authority and was conducted under Oregon Administrative Rule 333–019–0005 (Conduct of Special Studies).

## Results

### Patient Characteristics and Species Isolated

We identified 334 patients with extrapulmonary isolates, including 1 patient with 2 distinct infections. Overall, 176 (53%) patients were female, and the median age was 50 years (range 0.8–92 years) (Table). Half (n = 167, 50%) of patients had *M. avium/intracellulare* complex (MAC) infection, 129 (38.6%) RGM infection, and 21 (6.3%) *M. marinum* infection.

**Table:** Characteristics of 334 nontuberculous mycobacterium infections, Oregon, USA, 2007–2012*

Category	Infection site†					Total, N = 334
Skin/soft tissue, n = 197	Disseminated, n = 57	Lymph node, n = 28	Joint, n = 14	Other, n = 38
Annual incidence/100,000 population	0.9	0.2	0.1	0.1	0.2	1.5
Patient demographics						
Sex						
F	115 (58)	19 (33)	15 (54)	7 (50)	20 (52)	176 (53)
M	82 (42)	38 (67)	13 (46)	7 (50)	18 (48)	158 (47)
Median age, y (range)	51 (0.8–92)	41 (1–82)	44 (0.8–76)	70 (39–88)	61 (21–88)	51 (0.8–92)
HIV positive	8 (4)	34 (60)	5 (18)	0 (0)	3 (8)	50 (15)
Mycobacterium species						
Rapid-growing species	85 (43.1)	5 (8.8)	2 (7.1)	3 (21.4)	13 (34.2)	108 (32.3)
*M. chelonae/abscessus* complex	40 (20.3)	2 (3.5)	1 (3.6)	1 (7.1)	8 (21.1)	52 (15.6)
*M. fortuitum* complex	28 (14.2)	1 (1.8)	1 (3.6)	1 (7.1)	1 (2.6)	32 (9.6)
*M. chelonae*	17 (8.6)	2 (3.5)	0 (0)	1 (7.1)	4 (10.5)	24 (7.2)
Slow-growing species						
*M. avium/intracellulare* complex	70 (35.5)	45 (78.9)	23 (82.1)	9 (64.3)	20 (52.6)	167 (50)
*M. marinum*	20 (10.2)	0 (0)	0 (0)	0 (0)	1 (2.6)	21 (6.3)
*M. goodii*	5 (2.5)	0 (0)	1 (3.6)	0 (0)	0 (0)	6 (1.8)
*M. aubagnense*	1 (0.5)	1 (1.8)	0 (0)	0 (0)	1 (2.6)	3 (0.9)
*M. xenopi*	1 (0.5)	0 (0)	1 (3.6)	0 (0)	1 (2.6)	3 (0.9)
*M. alvei*	2 (1)	0 (0)	0 (0)	0 (0)	0 (0)	2 (0.6)
*M. neoaurum*	0 (0)	2 (3.5)	0 (0)	0 (0)	0 (0)	2 (0.6)
Other species‡	13 (6.6)	4 (7)	1 (3.6)	2 (14.3)	2 (5.3)	22 (6.6)

*Values are no. (%) patients except as indicated. Total excludes 13 *M. gordonae* isolates: 6 skin/soft tissue, 1 disseminated, 1 lymph node, 5 other. †Skin/soft tissue: wound, abscess, tissue, or exit catheter; disseminated: blood, bone marrow, cerebrospinal fluid, pericardial fluid, or peritoneal fluid; lymph node: lymph node or neck abscess; joint: synovial or joint fluid; other: urine, eye, sinus, or nasopharyngeal. ‡Other species were 8 unspeciated and 1 each of *M. asiaticum, M. branderi, M. brisbanense, M. heckeshornense, M. immunogenum, M. interjectum, M. kansasii, M. lentiflavum, M. llaterense, M. mucogenicum/phocaicum, M. obuense, M. simiae, M. thermoresistible, and M. vaccae*.

### Results by Site of Infection

Among the 334 extrapulmonary NTM infections, 197 (59.0%) were skin/soft tissue, 57 (17.1%) were disseminated, 28 (8.4%) were lymph node, 14 (4.2%) were joint, and 38 (11.4%) were other (Table). The overall species distribution was 50% MAC, 22.8% *M. chelonae/abscessus* complex or *M. chelonae*, 9.6% *M. fortuitum*, and 6.3% *M. marinum.* We identified an additional 13 patients with *M. gordonae* isolates. Patients with skin/soft tissue infections were more commonly female (58%), whereas disseminated infections occurred predominantly in male patients (67%); of these, 79% of patients had MAC infection. The median age of patients with disseminated infections was 41 years, and 60% of these infections occurred in HIV-positive patients. Lymph node infections were 82.1% MAC; 18% of these patients were HIV positive. Among 23 HIV-negative patients with lymph node infections, 10 (44%) were <5 years of age. Only 14 joint infections were reported; these patients had a median age of 70 years, and 64.3% of infections were caused by MAC. Of other infections, 52.6% were caused by MAC and 31.6% by *M. chelonae/abscessus* complex or *M. chelonae.*

### Estimates of Annual Incidence

The average annual incidence of extrapulmonary NTM infection during 2007–2012 was 1.5 (95% CI 1.1–1.8) cases/100,000 population. The incidence was 1.4 (CI 1.0–1.8) cases/100,000 population in 2007, peaked at 1.7 (CI 1.3–2.1)/100,000 in 2009, and decreased to 1.3 (CI 1.0–1.7)/100,000 in 2012 (Figure). The Poisson estimate of change in annual incidence was not significant, at −1.8% (95% CI −0.08 to 0.04; p = 0.6). The incidence by site of isolation was 0.9 cases/100,000 population for skin/soft tissue, 0.2/100,000 for disseminated, 0.2/100,000 for lymph, 0.1/100,000 for joint, and 0.2/100,000 other. The average annual incidence was 0.7 cases/100,000 population for MAC, 0.2/100,000 for *M. abscessus/chelonae* complex, and 0.1/100,000 each for *M. fortuitum*, *M. chelonae,* and *M. marinum*.

**Figure:**
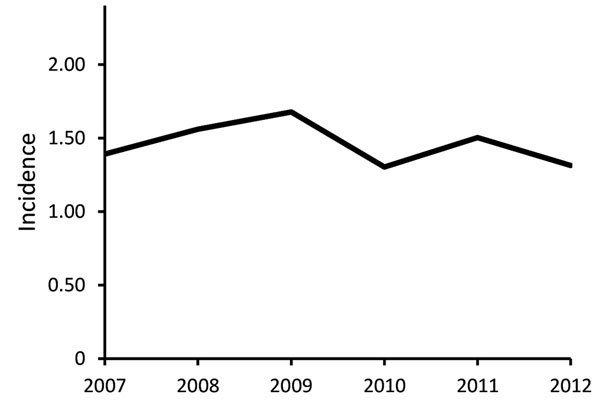
Observed incidence (cases/100,000 population) of extrapulmonary nontuberculous mycobacterium infection (excluding *Mycobacterium gordonae*), Oregon, USA, 2007–2012.

## Discussion

We describe Oregon’s population-based experience with extrapulmonary NTM infections before these infections were made reportable in 2014. The annual incidence remained stable over the study period, averaging 1.5 cases/100,000 population. In contrast with pulmonary NTM disease, which occurs predominantly in female patients, half of patients with extrapulmonary infections and two thirds of patients with disseminated NTM are male (*8*,*9*). A smaller proportion of extrapulmonary infections is caused by MAC than by pulmonary disease, and RGM cause 43.1% of skin/soft tissue infections. Overall, 15% of extrapulmonary infections occurred in HIV-positive patients.

Our observed overall average incidence was comparable to the annualized prevalence (1.6 cases/100,000 population) originally reported in Oregon during 2005–2006 (*10*). The skin/soft tissue prevalence/incidence in each time period was also identical at 0.9 cases/100,000 population, accounting for 58.9% of all extrapulmonary infections in our study. More recently, Smith et al. reported a higher prevalence of extrapulmonary NTM infection of 2.8 cases/100,000 population in 3 counties in North Carolina during 2006–2010 (*11*). Of 184 North Carolina patients with non–*M. gordonae* extrapulmonary NTM isolates, 51 (28%) were from a sterile site (equivalent to joint/disseminated/lymph node by our definition), 15 (8%) were dermal, 7 (4%) were catheter/implant related, and 111 (60%) were categorized as other. Because of their different classifications, it is difficult to compare results by site directly. However, in North Carolina, a similar proportion of extrapulmonary infections overall was caused by rapidly growing NTM (37%, compared with 31% in our study).

RGM are most commonly associated with skin/soft tissue infections. We observed a similar proportion of skin/soft tissue infections caused by RGM in Oregon compared with our prior study (43% vs. 51% in 2005–2006). Although describing small subsets of our skin/soft tissue infection category, a similarly or slightly higher proportion of dermal (10/15, 67%) and catheter/implant-related (2/7, 43%) extrapulmonary infections in North Carolina were caused by RGM (*11*). In our data, >80% of RGM *M. fortuitum* and slow-growing *M. goodii* and *M. marinum* infections were associated with skin/soft tissue infections.

Other categories of extrapulmonary NTM infections were less common. Disseminated infection, representing 17% of extrapulmonary infections, typically occurs in severely immunocompromised patients with AIDS (CD4+ counts <50), hematologic malignancies, or transplants (*12*,*13*). Positive HIV status was a notable contributor to infection in our study, associated with 60% of disseminated NTM. As reported previously, median annual incidence of disseminated NTM in Oregon during 2007–2012 in HIV-positive patients was high, at 110 cases/100,000 HIV person-years (*13*). Given the lower proportion of lymph, skin/soft tissue, or other infections with HIV, it is possible that some of these infections also represent disseminated infection in HIV patients.

In our data, only 43% of patients with pediatric lymphadenitis were <5 years of age, even after excluding those with HIV. Pediatric lymphadenitis occurs primarily in immunocompetent children <4 years of age, so this is an unusual pattern (*14*). Lymph node infections represented <10% of extrapulmonary infections. In the North Carolina study, only 3% of cases were isolated from the lymph node, although some may have been misclassified as neck infections (*11*). Of the 12 neck isolations in North Carolina, 8 were in children <3 years of age. In contrast, patients in our study with NTM infections of the joint (4% of total extrapulmonary infections), with a median age of 70, likely represent surgical site infections. Oregon previously investigated a cluster of 9 NTM infections involving joint prostheses occurring in 2013–2014 (*15*).

The strengths of this study include complete capture of extrapulmonary cases statewide over a 7-year period, allowing population-based analyses and analysis of trends. The disease incidence should be considered a minimal estimate, requiring the physician to order the appropriate diagnostic test (acid-fast bacillus culture). We were also able to link to the state HIV database and identify HIV-positive patients. Study limitations included a lack of clinical information to identify other underlying conditions and risk factors for infection. We were also unable to distinguish *M. chelonae* from *M. abscessus* in the *M. chelonae/abscessus* complex cases.

More detailed clinical data and exposure history for NTM infections in Oregon will be available in the future from state surveillance efforts, aiding in the identification of outbreaks that require public health intervention. However, it is likely that relatively few cases are associated with outbreaks. During the first 2 years of reportability in Oregon, only 11 (11%) of 98 extrapulmonary NTM isolates were linked epidemiologically (*16*). Reporting and follow-up of all patients with extrapulmonary isolates may be useful for detecting previously unidentified environmental sources of NTM, such as specific watersheds. Subspeciation and molecular typing of isolates may be necessary to identify clusters of more common species. Detailed follow-up on patients isolating *M. gordonae* to confirm whether it is the most likely cause of disease will inform whether or not to include it as a reportable infection along with other NTM species.

In conclusion, unlike pulmonary infections, which are increasing, extrapulmonary NTM incidence in Oregon is stable. Similar to pulmonary NTM, MAC causes most disseminated and lymph node infections. In contrast, RGM species are much more common causes of skin/soft tissue and other infections. Although the literature highlights clusters and outbreaks, most extrapulmonary NTM infections are likely isolated cases. Now that extrapulmonary NTM infections have been made reportable in Oregon (2014) and other states, we anticipate additional population-based estimates to be made available in the future.
